# Characterization of urinary cotinine in non-smoking residents in smoke-free homes in the Korean National Environmental Health Survey (KoNEHS)

**DOI:** 10.1186/s12889-016-3212-9

**Published:** 2016-07-11

**Authors:** Jeonghoon Kim, Kiyoung Lee

**Affiliations:** Department of Environmental Health, Graduate School of Public Health, Seoul National University, 1 Gwanak-ro, Gwanak-gu, Seoul 151-742 Republic of Korea; Department of Environmental Health Research, Seoul Medical Center, 156 Sinnae-ro, Jungnang-gu, Seoul 131-795 Republic of Korea; Institute of Health and Environment, Graduate School of Public Health, Seoul National University, 1 Gwanak-ro, Gwanak-gu, Seoul 151-742 Republic of Korea

**Keywords:** Cotinine, Housing type, Nonsmoker, Secondhand smoke, Smoke-free home

## Abstract

**Background:**

The objectives of this study were to determine urinary cotinine concentrations in non-smoking residents of smoke-free homes and to establish the relationship of urinary cotinine with housing type and other socio-demographic and secondhand smoke (SHS) exposure factors.

**Methods:**

We used data from the Korean National Environmental Health Survey I (2009–2011). The study included 814 non-smoking adult residents living in apartments, attached, and detached housing. Residents who lived with smokers were excluded. Urinary cotinine concentration was used as a biomarker for SHS exposure. The factors associated with urinary cotinine levels in non-smoking residents were determined using multivariate regression analysis.

**Results:**

Urinary cotinine was detected in 88 % of the 814 non-smoking residents of smoke-free homes. The urinary cotinine concentrations of residents living in attached [1.18 ng/mg creatinine (Cr)] and detached housing (1.23 ng/mg Cr) were significantly higher than those of residents who lived in apartments (0.69 ng/mg Cr). Urinary cotinine concentrations were significantly higher in residents who were men, those with a household income ≤1000 USD/month, those who were former smokers with >1 year and ≤1 year of not smoking, and those who experienced SHS odor every day. In the multivariate regression analysis, housing type, sex, former smoking status, and frequency of experiencing SHS odor were associated with urinary cotinine concentrations (*R*^2^ = 0.14).

**Conclusions:**

The majority of non-smoking residents of smoke-free homes had detectable urinary cotinine. Housing type, sex, former smoking status, and frequency of experiencing SHS odor were predictors for urinary cotinine concentrations in the study participants.

## Background

Secondhand smoke (SHS) contains more than 7,000 chemicals, including more than 69 known carcinogens [1]. It is associated with cardiovascular disease, coronary heart disease, asthma, other respiratory symptoms, and lung cancer [2–5]. Epidemiological studies have reported that SHS exposure is causally linked with increasing morbidity and mortality [4]. SHS was estimated to have caused 603,000 premature deaths in 192 countries in 2004, corresponding to about 1 % of worldwide mortality [6].

Based on mounting scientific evidence of the adverse health effects of SHS exposure, many countries have implemented smoke-free regulations in public indoor areas and workplaces, which have led to significant reductions in SHS exposure and positive health effects [7–9]. However, the home environment has remained a significant source of SHS exposure [4, 10].

Many studies that related to SHS exposure at home have focused on nonsmoking residents who were living with smokers. Recently, SHS exposure in smoke-free multiunit housing (MUH) has increased attention. The residents can be exposed to SHS because SHS from MUH with residents who smoke can be transferred to neighboring units [11, 12]. In a 2009 survey in the US, 25.8 % (79.2 million) lived in MUH, and 62.7 million MUH residents followed smoke-free home rules [13]. Of those residents, SHS incursions were reported in 44.0–46.2 % of the residences. In Denmark, 28.2 % of MUH residents who lived in homes where no one smoked inside were reported that neighbor smoke seeped into their homes from other places (e.g., other unit, stairway, etc.) [14]. Because people tend to spend a large proportion of their time indoors in their homes, SHS exposure in residences can be a significant contributor to their total exposure. In the US, people spend about 69 % of their time in their home [15]. This compares with a daily mean of 59 % in Korea [16].

Limited studies assessed the SHS biomarker levels of non-smokers living in smoke-free homes. Cotinine, a metabolite of nicotine, is a specific and sensitive biomarker of SHS exposure [17]. It can be measured in urine, whole blood, serum, plasma, and saliva, and has an average half-life of 16 h. One study that assessed the blood serum cotinine levels of US children who lived in homes where no one smoked indoors reported higher serum cotinine concentrations in children who lived in apartments than in those living in detached residences [18].

In the present study, urinary cotinine concentration data from the Korean National Environmental Health Survey (KoNEHS) I, conducted by the National Institute of Environmental Research and the Ministry of Environment as a national bio-monitoring program, were used. The objectives of this study were to determine the urinary cotinine concentrations of non-smoking residents living in smoke-free homes and to establish the relationship of urinary cotinine concentration with housing type and other socio-demographic and SHS exposure factors.

## Methods

### Selection of data and study variables

KoNEHS I (2009–2011) used a stratified cluster sampling design that took into consideration geographic and socio-economic factors based on the household surveys of the 2005 Population and Housing Census. Overall, 6,311 individuals who were older than 19 years of age participated in KoNEHS I. The survey collected participants’ questionnaires and blood and urine samples.

The 2009 data from KoNEHS (*n* = 2,101) were excluded because the questionnaires in that year did not ask whether the subjects resided with smokers in their homes. The study included subjects who lived in apartments, attached, and detached house. An apartment was defined as a high-rise multifamily building (≥5 stories) which was often owned by occupants. An attached house was a multi-family house (≤4 stories) with multiple owners. A detached house included single-family and multifamily house (≤3-story) with one owner. Other types of housing, such as non-residential buildings, were excluded due to their small numbers.

Data from the questionnaires were restricted to the following respondents: those who (1) lived in apartments, attached, or detached housing (*n* = 4,122); (2) had never smoked or were former smokers (*n* = 3,161); (3) were not living with smokers in their homes (*n* = 2,026); (4) spent their time mainly at home indoors (*n* = 933); (5) reported household income (n = 923); and (6) reported the frequency or duration of SHS odor in their homes (*n* = 922). Of these 922 residents, those who were suspected to be smokers (*n* = 26) based on their urinary cotinine concentrations (≥100 ng/ml) [19] and those whose creatinine concentrations were not estimated were excluded (*n* = 82). Ultimately, samples from 814 individuals were included for further analysis. This study was approved by the Institutional Review Board of the National Institute of Environmental Research and the Ministry of the Environment (IRB number: EED-354).

Other socio-demographic data, such as sex (men/women), age (19–39 years, 40–59 years, and ≥60 years), education (middle school or less, high school, and college or higher), household income (≤1000 USD/month, 1001–2000 USD/month, 2001–3000 USD/month, and >3000 USD/month), and former smoking status (never smoked, >1 year not smoking, and ≤1 year not smoking) were included. Household income was classified based on 0–25, 26–50, 51–75, and 76–100 percentiles of the 814 subjects. SHS exposure factors, such as frequency of experiencing SHS odor (none, 1–2 times/week, 3–6 times/week, and every day) and duration of experiencing SHS odor (none, 1–5 min/day, 6–30 min/day, and ≥31 min/day) were included.

### Urinary cotinine

Spot urine samples (80 ml) were collected mid-urination and frozen at −20 °C until analysis. All urinary cotinine analyses were carried out at an analytical laboratory certified by the Korean Ministry of Health and Welfare. For urinary cotinine analysis, internal standard (diphenylamine), 50 μL of 0.1 M sodium hydroxide, and 500 μL of chloroform was added to 1 mL of urine samples. After the solution was centrifuged, sodium sulfate was added to remove waters. The specific method of urinary cotinine analysis has been described in a previous study [20]. The urinary cotinine concentrations were measured using gas chromatograph-mass spectrometry with a Clarus 600 T (PerkinElmer, Turku, Finland). The method detection limit (MDL) for urinary cotinine was 0.27 ng/ml. Urinary cotinine concentrations below the MDL were assigned as 0.2 ng/ml (MDL/√2). Urinary creatinine was determined with an alkaline picrate kinetic (Jaffe) method using an Adiva 2400 Chemistry System (Siemens Healthcare Diagnostics). All urinary cotinine concentrations were adjusted by the urinary creatinine concentrations.

### Statistical analysis

SAS 9.2 (SAS Institute, Inc., Cary, NC, USA) was used for all statistical analyses and calculations. The sample weights were used in all analyses in a stratified cluster sampling design. The proportions of variables by housing type were calculated with SAS PROC SURVEYFREQ. Because the urinary cotinine concentrations were skewed, the natural log (ln)-transformations of the urinary cotinine concentrations were used for all analyses. The geometric mean (GM) and 95 % confidence interval (CI) of the urinary cotinine concentrations by variable were calculated with SAS PROC SURVEYMEANS. SAS PROC SURVEYREG was used to perform a univariate and multivariate linear regression to assess the associations between urinary cotinine concentrations and variables. Variables with *p*-values <0.05 in the univariate analysis were included in the multivariate regression analysis. A *p*-value of 0.05 was considered significant in all analyses.

## Results

The characteristics of the study population by housing type are shown in Table 1. Non-smoking residents of detached housing represented slightly less than half (43.8 %) of the overall study population, and residents of apartments and attached housing comprised 36.9 % and 19.2 % of the study subjects, respectively. The majority of the participants was men (64.0 %), 40–59 years of age (34.1 %), had middle school or less education (40.5 %), and had household incomes of 1001–2000 USD/month (28.1 %). Former smokers with >1 year of not smoking and ≤1 year of not smoking comprised 19.5 % and 4.2 % of the subjects, respectively. The percentages of residents who experienced SHS odor ≥1 time/week or ≥1 min/day in apartments, attached, and detached housing, were 20.7 %, 16.1 %, and 14.6 %, respectively.

**Table 1:** Socio-demographic and SHS exposure factors among non-smoking residents of smoke-free homes by housing type^a^

	Apartment	Attached housing	Detached housing	Total
	*%* (*n*)	*%* (*n*)	*%* (*n*)	*%* (*n*)
Total	36.9 (287)	19.2 (131)	43.8 (396)	100.0 (814)
Sex				
Men	71.9 (204)	63.2 (82)	57.6 (213)	64.0 (499)
Women	28.1 (83)	36.8 (49)	42.4 (183)	36.0 (315)
Age (year)				
19–39	34.3 (79)	27.0 (32)	12.7 (33)	23.5 (144)
40–59	41.7 (135)	37.1 (50)	38.0 (159)	39.2 (344)
≥ 60	24.0 (73)	35.8 (49)	49.3 (204)	37.3 (326)
Education				
Middle school or less	21.2 (69)	33.7 (54)	59.7 (254)	40.5 (377)
High school	43.8 (117)	34.9 (41)	25.5 (100)	34.1 (258)
College or higher	35.0 (101)	31.4 (36)	14.8 (42)	25.4 (179)
Household income (USD/month)				
≤ 1000	7.3 (27)	25.9 (36)	38.7 (157)	24.6 (220)
1001–2000	24.6 (66)	29.7 (40)	30.4 (113)	28.1 (219)
2001–3000	31.3 (91)	21.4 (29)	17.5 (75)	23.4 (195)
> 3000	36.7 (103)	23.0 (26)	13.4 (51)	23.8 (180)
Former smoker				
Never smoked	83.4 (241)	71.8 (95)	72.4 (275)	76.3 (611)
>1 year not smoking	15.0 (43)	19.7 (26)	23.2 (102)	19.5 (171)
≤1 year not smoking	1.6 (3)	8.6 (10)	4.4 (19)	4.2 (32)
Frequency of SHS odor (times/week)				
None	79.3 (231)	83.9 (106)	85.4 (333)	82.8 (670)
1–2	10.5 (35)	8.6 (14)	7.2 (30)	8.7 (79)
3–6	6.2 (11)	4.2 (5)	3.6 (14)	4.7 (30)
Every day	4.0 (10)	3.3 (6)	3.8 (19)	3.8 (35)
Duration of SHS odor (min/day)				
None	79.3 (231)	83.9 (106)	85.4 (333)	82.8 (670)
1–5	9.9 (28)	5.8 (10)	3.7 (18)	6.4 (56)
6–30	4.6 (12)	9.5 (12)	5.5 (22)	6.0 (46)
≥ 31	6.2 (16)	0.8 (3)	5.4 (23)	4.8 (42)

^a^All estimated data are based on weighted analyses

Urinary cotinine was detected in 88 % of the 814 residents. The GM of the urinary cotinine concentrations was 0.98 ng/mg creatinine (Cr) (95 % CI: 0.84–1.14). In the univariate analysis, several variables were significantly associated with urinary cotinine concentrations (Table 2). The urinary cotinine concentrations of residents who lived in attached (GM: 1.18 ng/mg Cr; 95 % CI: 0.91–1.52) and detached (GM: 1.23 ng/mg Cr; 95 % CI: 0.96–1.58) housing were significantly higher than those of residents who lived in apartments (GM: 0.69 ng/mg Cr; 95 % CI: 0.55–0.86). The GM of the urinary cotinine concentrations of residents were 1.56 ng/mg Cr (95 % CI: 1.24–1.96) for men and 0.76 ng/mg Cr (95 % CI: 0.64–0.90) for women; these values were significantly different. The urinary cotinine concentrations of residents who had a household income of ≤1000 USD/month (GM: 1.21 ng/mg Cr; 95 % CI: 0.92–1.59) were significantly higher than those of residents with a household income of 2001–3000 USD/month (GM: 0.81 ng/mg Cr; 95 % CI: 0.62–1.07). The urinary cotinine concentrations of former smokers with >1 year of not smoking (GM: 1.70 ng/mg Cr; 95 % CI: 1.27–2.27) and ≤1 year of not smoking (GM: 3.91 ng/mg Cr; 95 % CI: 2.32–6.60) were significantly higher than the concentrations of residents who had never been smokers (GM: 0.79 ng/mg Cr; 95 % CI: 0.68–0.93). The urinary cotinine concentrations of residents who experienced SHS odor every day (GM: 2.07 ng/mg Cr; 95 % CI: 1.11–3.88) were significantly higher than those of residents who never experienced SHS odor (GM: 0.93 ng/mg Cr; 95 % CI: 0.79–1.10). However, urinary cotinine concentrations were not significantly associated with age, education, or duration of experiencing SHS odor.

**Table 2:** Urinary cotinine concentrations (ng/mg creatinine) and univariate analysis for natural log-transformed cotinine concentrations of non-smoking residents of smoke-free homes^a^

	GM (95 % CI)	*β*	Standard error	*p*-value
Type of housing				
Apartment	0.69 (0.55–0.86)	Reference		
Attached housing	1.18 (0.91–1.52)	0.54	0.17	0.002
Detached housing	1.23 (0.96–1.58)	0.58	0.17	<0.001
Sex				
Men	1.56 (1.24–1.96)	Reference		
Women	0.76 (0.64–0.90)	−0.72	0.13	<0.0001
Age (year)				
19–39	0.80 (0.60–1.06)	Reference		
40–59	1.09 (0.90–1.32)	0.32	0.17	0.070
≥ 60	1.01 (0.80–1.27)	0.24	0.19	0.219
Education				
Middle school or less	1.09 (0.87–1.37)	Reference		
High school	0.89 (0.70–1.14)	−0.20	0.17	0.220
College or higher	0.95 (0.70–1.28)	−0.14	0.19	0.436
Household income (USD/month)				
≤ 1000	1.21 (0.92–1.59)	Reference		
1001–2000	1.02 (0.80–1.31)	−0.16	0.16	0.297
2001–3000	0.81 (0.62–1.07)	−0.39	0.20	0.045
> 3000	0.91 (0.69–1.21)	−0.28	0.21	0.175
Former smoker				
Never smoked	0.79 (0.68–0.93)	Reference		
> 1 year not smoking	1.70 (1.27–2.27)	0.76	0.15	<0.0001
≤ 1 year not smoking	3.91 (2.32–6.60)	1.60	0.27	<0.0001
Frequency of SHS odor (times/week)				
None	0.93 (0.79–1.10)	Reference		
1–2	0.99 (0.71–1.37)	0.05	0.17	0.762
3–6	1.33 (0.83–2.13)	0.35	0.24	0.140
Every day	2.07 (1.11–3.88)	0.80	0.33	0.017
Duration of SHS odor (min/day)				
None	0.93 (0.79–1.10)	Reference		
1–5	1.17 (0.72–1.91)	0.23	0.27	0.395
6–30	1.32 (0.90–1.94)	0.35	0.21	0.095
≥ 31	1.30 (0.78–2.20)	0.33	0.25	0.185

^a^All estimated data are based on weighted analyses

A multivariate regression analysis of ln-transformed urinary cotinine concentrations was performed using the significant variables identified in the univariate analysis. Housing type, sex, former smoking status, and frequency of experiencing SHS odor were associated with urinary cotinine concentrations (*R*^2^ = 0.14; Table 3). The urinary cotinine concentrations of residents who lived in attached and detached housing were significantly higher than the concentrations of those living in apartments. The urinary cotinine concentrations of residents who were men were marginally higher than those who were women. The urinary cotinine concentrations of former smokers with >1 year of not smoking and ≤1 year of not smoking were significantly higher than those of subjects who had never smoked. The urinary cotinine concentrations of residents who experienced SHS odor every day were marginally higher than the concentrations of those who never experienced SHS odor.

**Table 3:** Multivariate analysis for natural log-transformed urinary cotinine concentrations of non-smoking residents of smoke-free homes^a^

	*β*	Standard error	*p*-value
Type of housing			
Apartment	Reference		
Attached housing	0.39	0.17	0.011
Detached housing	0.44	0.17	0.024
Sex			
Men	Reference		
Women	−0.28	0.16	0.074
Household income (USD/month)			
≤ 1000	Reference		
1001–2000	−0.06	0.16	0.706
2001–3000	−0.17	0.20	0.384
> 3000	−0.06	0.20	0.769
Former smoker			
Never smoked	Reference		
> 1 year not smoking	0.47	0.17	0.007
≤ 1 year not smoking	1.24	0.28	<0.001
Frequency of SHS odor (times/week)			
None	Reference		
1–2	−0.07	0.17	0.655
3–6	0.13	0.20	0.520
Every day	0.60	0.31	0.053

R-squared values from the multivariate regression model were 0.14 after adjusting for all associated factors listed in the table

^a^All estimated data are based on weighted analyses

## Discussion

The urinary cotinine concentrations of the non-smoking residents living in smoke-free homes were significantly associated with housing type. The concentrations of non-smoking subjects living in attached and detached housing were 1.7- and 1.8-fold higher, respectively, than the concentrations of those living in apartments. This indicated that non-smoking residents living in attached and detached housing were more likely to be exposed to SHS than were those living in apartments.

Our findings on housing type differ from those of a study of serum cotinine concentrations in non-smoking children in the US living in homes where no one smoked inside the home [18]. The serum cotinine concentrations of non-smoking children who lived in apartments (0.075 ng/ml) were significantly higher than the concentrations of those living in attached (0.053 ng/ml) and detached housing (0.031 ng/ml). These differences are likely due to the different rates of residents who resided with smokers by housing type between Korea and America. The US children who resided with smokers inside the home were more likely to live in apartments than in detached housing [18]. In Korea, data from KoNEHS I showed that the non-smokers residing with smokers were less likely to live in apartments (49.2 %) than in attached (59.1 %) and detached housing (53.1 %).

The housing type reflected the socio-economic status of the residents. A previous study reported that socio-economic status was associated with SHS exposure among non-smoking residents [10]. Plasma cotinine concentrations among non-smoking adults were higher in those who were living in more socio-economically disadvantaged circumstances, suggesting that non-smoking residents of higher socio-economic level have lower SHS exposure. This trend was similar to our findings of lower urinary cotinine concentrations in non-smoking residents living in apartments. In Korea, apartments are high-rise multifamily buildings that are similar to high-rise condominium buildings in the USA. Residents living in apartments tended to be of a higher socio-economic level than those living in other housing types.

Possible sources of SHS in smoke-free homes include SHS incursion from neighboring units and from outside the building. Evidence of SHS incursion from smoking units was reported in 2 of 14 smoke-free units and 6 of 8 hallways inside 11 MUH buildings [11]. Temporal profiles of concentrations of particulate matter smaller than 2.5 μm in diameter (PM_2.5_), an airborne marker for SHS exposure [21], demonstrated that PM_2.5_ concentrations in hallways increased instantly when the front door of a smoking unit was opened, and later the PM_2.5_ concentrations in nearby smoke-free units increased. Outdoor tobacco smoke near building entrances has been shown to drift into indoor spaces [22]. Median PM_2.5_ concentrations were 17.2 μg/m^3^ in outdoor main entrances where smoking occurred and 18.2 μg/m^3^ in halls adjacent to outdoor areas. However, the median PM_2.5_ concentrations in outdoor and indoor areas with no presence of specific PM_2.5_ sources (controls) were 13.0 μg/m^3^ and 10.4 μg/m^3^, respectively.

Non-smoking residents in smoke-free homes may also be exposed to residual tobacco smoke pollution. The SHS pollutant can remain on dust and surfaces in the indoor environment and be re-suspended and/or re-emitted into the air or react with other compounds to produce secondary pollutants, which are referred to as third-hand smoke (THS) [23]. Dust and surface nicotine concentrations in homes previously occupied by smokers decreased after non-smoking residents moved in, but were still seven- to eight-fold higher than in previously smoke-free homes [24]. Average urinary cotinine concentrations of the youngest residents from former smokers’ homes (*n* = 5; 0.61 ng/ml) were higher than the concentrations of those from former smoke-free homes (*n* = 13; 0.13 ng/ml¸ *p* = 0.12).

In addition to housing type, residents who were men had slightly higher urinary cotinine concentrations than residents who were women in the multivariate analysis. This finding is similar to that for serum cotinine concentrations measured in a non-smoking population in the US [25], in which the serum cotinine concentrations of non-tobacco users who were >17-year-old men were significantly higher than those of equivalent women. Another study reported significantly different GMs for urinary cotinine concentrations in non-smokers in Korean population, with values of 1.43 ng/ml (95 % CI: 1.29–1.57) for men and 1.16 ng/ml (95 % CI: 1.07–1.25) for women in 2011 [20]. A possible reason for the higher urinary cotinine concentrations in men is that men might be more likely to be exposed to SHS during social activities and in public places than are women [25], although the present study investigated residents who spent the majority of their time at home indoors.

Former smoking was significantly associated with higher urinary cotinine concentrations in the multivariate analysis. Former smokers of >1 year of not smoking and ≤1 year of not smoking had 2.2- and 4.9-fold higher urinary cotinine concentrations, respectively, than did subjects who had never smoked. The higher urinary cotinine concentrations in former smokers may be because former smokers who quit smoking recently might be smoked occasional cigarettes. The findings of this study are similar to those of a study that measured saliva cotinine concentrations in 97 non-smoking adults [26]. In that study, although the saliva cotinine concentrations of former smokers and adults who had never smoked were not significantly different, the median concentrations of salivary cotinine were slightly higher in former smokers (2.8 ng/ml) than in subjects who had never smoked (2.4 ng/ml; *p* = 0.87). The median cotinine concentrations of former smokers of ≤6 month of not smoking (9.5 ng/ml) were significantly higher than the concentrations of former smokers of >6 month of not smoking (2.2 ng/ml; *p* = 0.02).

Daily experience of SHS odor in the home was slightly associated with increased urinary cotinine concentrations in the multivariate analysis. Although this was not statistically significant, urinary cotinine concentrations increased with increasing frequency of experiencing SHS odor. The relationship between urinary cotinine levels and frequency of experiencing SHS odor differed by housing type. The urinary cotinine concentrations of non-smoking residents in detached housing were highest, followed by those of non-smoking residents in attached housing and apartments. However, non-smoking residents who experienced SHS odor ≥1 time/week were lowest in detached housing followed by attached housing and apartments. The self-reported perception of SHS exposure may have differed according to the social tolerance level. When smoking prevalence rates are high, SHS exposure may be less likely to be perceived. Data from KoNEHS I showed that rates of non-smokers residing with smokers were more likely to live in attached and detached housing than in apartment. These findings indicate that non-smoking residents of detached housing may have higher tolerance regarding SHS exposure than the residents of other housing types.

This study has several limitations. Because detached houses in the present study included single-family and multifamily houses, urinary cotinine concentrations of non-smoking residents in smoke-free home were not separately assessed these types of houses. The KoNEHS I applied the housing types because it was legal classification. In future, it is needed to precise classification of housing types to assess urinary cotinine concentrations of non-smoking residents who lived in smoke-free single family and multifamily houses.

Although we selected non-smoking residents not living with smokers in their homes, they might have been exposed to SHS in their homes from non-resident smokers. Although this study selected residents who spent the majority of their time at home indoors, they were possibly exposed to SHS outside their homes, such as outdoors, in transportation, and at social venues. This could not be assessed in this study, since the questionnaire did not ask whether regular guests or visitors smoked in the homes or residents were exposed to SHS exposure outside homes. The study could not account for nicotine containing products, including e-cigarettes and nicotine replacement therapies (e.g. nicotine patch) among former smokers. These unmeasured factors may have contributed to the urinary cotinine concentrations.

Previous studies have quantified SHS exposure in smoke-free homes using environmental markers (e.g., particulate matter, volatile organic compounds, etc.) [11, 12]. The present study measured urinary cotinine concentrations in non-smoking residents living in smoke-free homes to determine SHS exposure and associated factors. The findings are based on nationwide survey data. Although the frequency of SHS odor in smoke-free homes was positively associated with the urinary cotinine concentrations of non-smoking residents, the sources of tobacco smoke pollutants were not identified. Further study is needed to identify the sources of SHS in smoke-free homes in Korea.

## Conclusions

Data from KoNEHS I were used to determine the urinary cotinine concentrations of 814 non-smoking residents of smoke-free homes. High detection rate of urinary cotinine in the non-smoking residents suggested that the most non-smoking residents in Korea might be exposed to secondhand smoke. The urinary concentration was associated with housing type, sex, former smoking status, and frequency of experiencing SHS odor in the home. The findings suggested that residents in smoke-free homes might be exposed to SHS from incursion from neighboring units and from outside the building, as well as from THS in the homes. Contribution of each exposure pathway in residence may be needed for better protection.

## Abbreviations

CI, confidence interval; Cr, creatinine; KoNEHS, Korean National Environmental Health Survey; MDL, method detection limit; GM, geometric mean; MUH, multiunit housing; PM_2.5_, particulate matter smaller than 2.5 μm in diameter; SHS, secondhand smoke; THS, Third-hand smoke
